# Role of iron chelation in hemorrhagic myocardial infarction: a quantitative CMR study

**DOI:** 10.1186/1532-429X-14-S1-P20

**Published:** 2012-02-01

**Authors:** Nilesh R Ghugre, Jennifer Barry, John C Wood, Alan Moody, Bradley H Strauss, Graham Wright

**Affiliations:** 1 Imaging Research, Sunnybrook Research Institute, Toronto, ON, Canada; 2 Division of Cardiology, Childrens Hospital Los Angeles, Los Angeles, CA, USA; 3 Department of Medical Imaging, Sunnybrook Health Sciences Centre, Toronto, ON, Canada; 4 Schulich Heart Program, Sunnybrook Health Sciences Centre, Toronto, ON, Canada; 5 Department of Medical Biophysics, University of Toronto, Toronto, ON, Canada

## Summary

Reperfusion hemorrhage is an independent predictor of adverse left-ventricular remodeling following acute myocardial infarction. Iron chelation may potentially alleviate the toxic and pro-inflammatory effects of iron degradation products. CMR can provide insights into the interaction between hemorrhage and iron chelator.

## Background

It has been speculated that iron chelation may be beneficial in acute myocardial infarction (AMI) and that early treatment can limit ischemia-reperfusion injury and also reduce infarct size. However, the role of iron chelation in hemorrhagic myocardial infarction, where it would be most suited, has not yet been explored. Reperfusion hemorrhage results in accumulation of iron degradation products of hemoglobin that may be pro-inflammatory as free iron is toxic in nature. The purpose of the study was to investigate the interaction between iron chelating agent deferiprone (DFP) and hemorrhage in a porcine model of myocardial infarction and monitor remodeling by cardiovascular magnetic resonance (CMR).

## Methods

The study involved two groups of animals that were subjected to a 90 min balloon occlusion of the LAD followed by reperfusion - untreated (N=2) and DFP treated (N=2). DFP (supported by ApoPharma Inc., Toronto, ON) was administered (orally) a few hours before the procedure (pre-loading) and treatment was continued with a daily dose of 100 mg/kg. Imaging was performed on a 3T MRI scanner (MR 750, GE Healthcare) pre-AMI (healthy) and day 2-week 4 post-AMI. Edema was evaluated by T2 quantification using a T2-prepared spiral sequence and hemorrhage was identified by T2* determined using a multi-echo gradient-echo acquisition. Infarct assessment was performed by delayed hyperenhancement (DHE) using an IR-GRE sequence.

## Results

**Figure 1:**
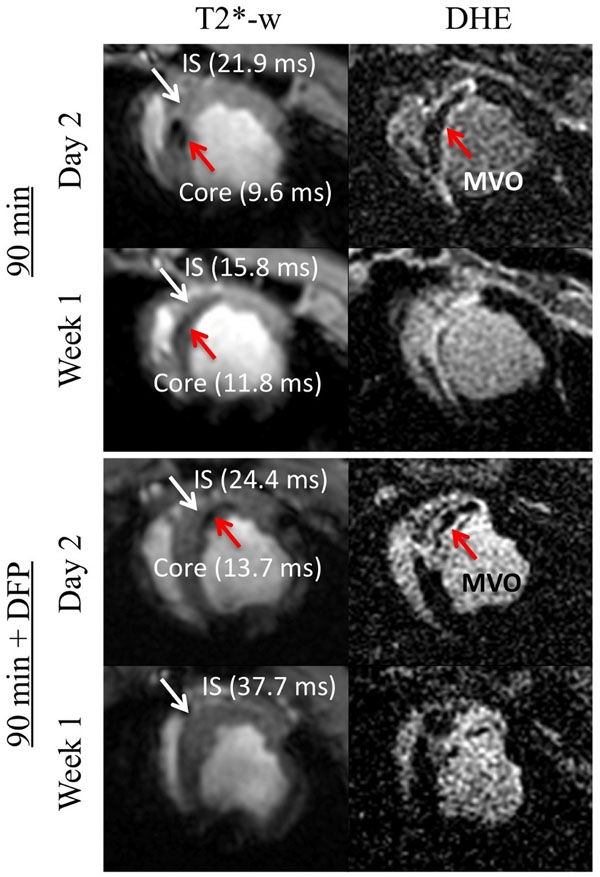
Short axis images from a representative animals subjected to 90 min LAD occlusion without (top panel) and with (bottom panel) treatment of iron chelator deferiprone (DFP). In the DFP group, hemorrhage, as indicated by T2* image (red arrows) was observed only on day 2 but resolved by week 1, unlike the untreated group where hemorrhage persisted even at week 1. The iron neutralizing capacity of DFP is apparent from this example. In both groups, microvascular obstruction (MVO) was seen on day 2 that was partially resolved by week 1.

**Figure 2:**
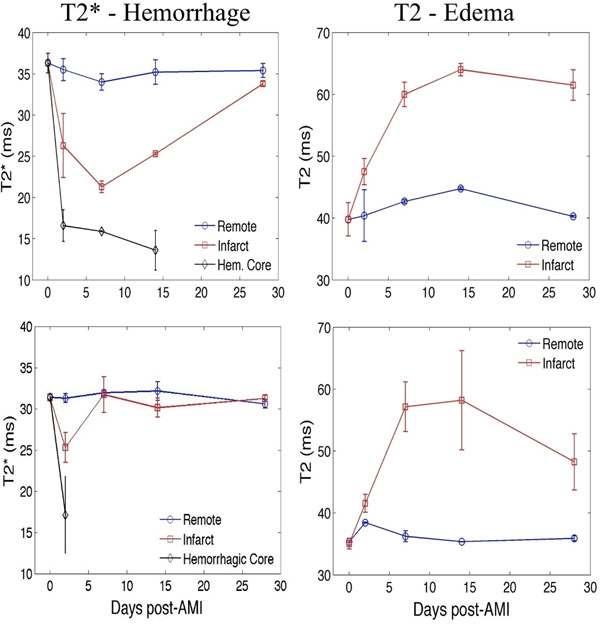
Cumulative time course of T2 and T2* parameters post-AMI pooled across all animals (N=2) in the 90 min infarct - untreated (top panel) and treated with deferiprone (DFP, bottom panel); error bars show standard error and day 0 indicates control MRI scans in healthy animals. Infarct zone is shown in red; remote myocardium is shown in blue; hemorrhagic core is shown in black.

Figure 1 demonstrates representative images from the two groups while Figure 2 shows the cumulative time course of the CMR measurements. In the DFP group, hemorrhage was observed only on day 2 and by week 1 it had completely resolved. This was in contrast to the untreated group where resolution of hemorrhage was delayed to week 4. With DFP, inflammation or edema was substantially reduced by week 4 with T2 values approaching control levels. In the untreated group, edema persisted up to week 4. Ejection fraction (EF) was depressed by week 4 in both groups. However, end-diastolic and end-systolic volumes were relatively unchanged in the DFP group while they increased significantly in the untreated group.

## Conclusions

Hemorrhage may be a source of iron toxicity and a mediator of inflammation, directly contributing to adverse remodeling in the setting of AMI. DFP was able to penetrate the infarct zone and was also effective in neutralizing hemorrhagic byproducts. Elimination of hemorrhage resulted in faster resolution of edema and normal ventricular volumes, representing a beneficial remodeling process. Iron chelation could potentially serve as an adjunctive therapy in hemorrhagic AMI.

## Funding

We would like to acknowledge funding support from the Ontario Research Fund, the Canadian Institutes of Health Research and GE Healthcare.

